# Carbonation Kinetics of Ca(OH)_2_ Under Conditions of Entrained Reactors to Capture CO_2_

**DOI:** 10.1021/acs.iecr.1c04888

**Published:** 2022-02-24

**Authors:** B. Arias, Y. A. Criado, B. Pañeda, J. C. Abanades

**Affiliations:** INCAR-CSIC, C/ Francisco Pintado Fe No. 26, 33011 Oviedo, Spain

## Abstract

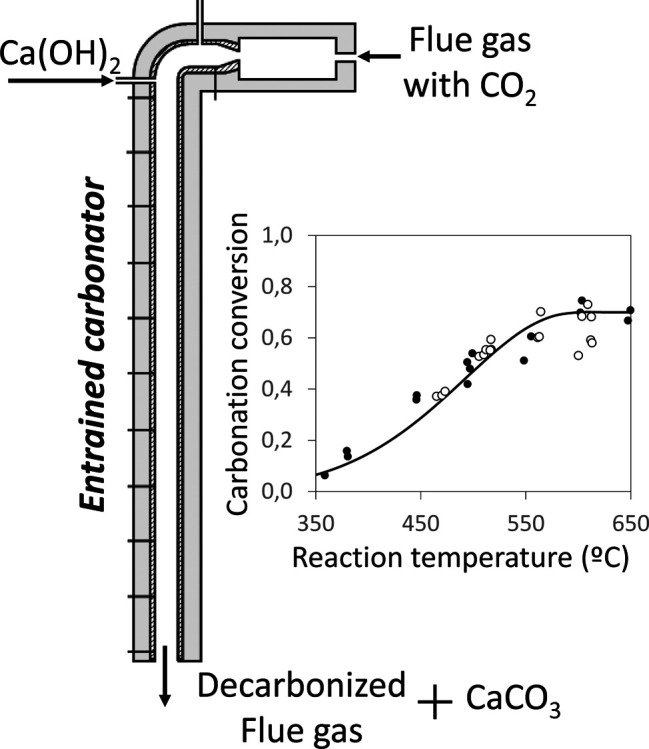

The use of Ca(OH)_2_ as a CO_2_ sorbent instead of CaO in calcium looping systems has the advantage of a much faster reaction rate of carbonation and a larger conversion degree to CaCO_3_. This work investigates the carbonation kinetics of fine Ca(OH)_2_ particles (<10 μm) in a range of reaction conditions (i.e., 350–650 °C and CO_2_ concentrations up to 25%_v_) that could be of interest for applications where a short contact time is expected between the solids and the gases (i.e., entrained bed carbonator reactors). For this purpose, experiments in a drop tube reactor with short reaction times (i.e., below 6 s) have been carried out. High carbonation conversions up to 0.7 have been measured under these conditions, supporting the viability of using entrained carbonator reactors. The experimental results have been fitted to a shirking core model, and the corresponding kinetic parameters for the carbonation reaction have been determined.

## Introduction

Postcombustion CO_2_ capture based on calcium looping (CaL) has been mainly developed in the last 20 years for standard power plant applications.^1−3^ This work is mainly concerned with the use of Ca(OH)_2_ in CaL systems, to exploit the enhanced reactivity and CO_2_ capture capacity of this material with respect to CaO. The hydration of CaO to produce Ca(OH)_2_ has been widely studied as a method to reactivate CaO sorbents in standard CaL systems and to revert the decay of activity with the number of carbonation calcination cycles.^2,4−12^ More recently, backup power systems combining CaL and extensive intermediate storage of CaO or Ca(OH)_2_^13−17^ have proposed the use of a compact entrained or fast bed reactors as carbonators, which demand for the high reactivity and high CO_2_-carrying capacity of the sorbent characteristic of Ca(OH)_2_.

Most of the previous studies on Ca(OH)_2_ at particle level have been focused on the use of partially reactivated sorbent under standard postcombustion CaL conditions in fluidized bed carbonators (i.e., temperatures of 650 °C, reaction times of a few minutes, and particle sizes above 100 μm). A few works have studied the use of Ca(OH)_2_ fine powder materials, resulting from the complete hydration of CaO, demonstrating that this sorbent can achieve conversions above 0.6 in a few seconds at temperatures around 650 °C with typical coal flue gas composition with CO_2_ concentrations around 15%_v_.^18−20^ Such reaction rates are 2 orders of magnitude faster than the parent CaO materials and can have important benefits in some new CaL systems using entrained reactors, similar to those used for in-duct sorbent desulfurization applications. We are particularly interested in systems to capture CO_2_ from the low concentration flue gases, such as those emitted from gas turbines (∼4%_v_ CO_2_), where there are equilibrium limitations in terms of CO_2_ capture efficiencies. Reaction temperatures below 600 °C are needed to access capture efficiencies over 90% (*p*_CO_2_eq at 600°C_ = 0.4%_v_) or even temperatures below 500 °C (*p*_CO_2_eq at 500°C_ = 0.02%_v_) for “CO_2_ polishing” applications involving capture efficiencies >99%. Low carbonation temperatures are known to yield modest carbonation conversions of CaO at temperatures below 600 °C,^21^ but the information is scarce for Ca(OH)_2_. Furthermore, there is a need to investigate the kinetics of carbonation of Ca(OH)_2_ powders in the range of temperatures and CO_2_ concentrations expected in the new applications. The objective of this work is to investigate the carbonation reaction kinetics of Ca(OH)_2_ in a drop tube reactor under relevant conditions (i.e., short gas–solid contact times, fine powders, suitable CO_2_ gas concentrations, etc.) for entrained bed carbonator reactors.

## Experimental Section

**Figure 1:**
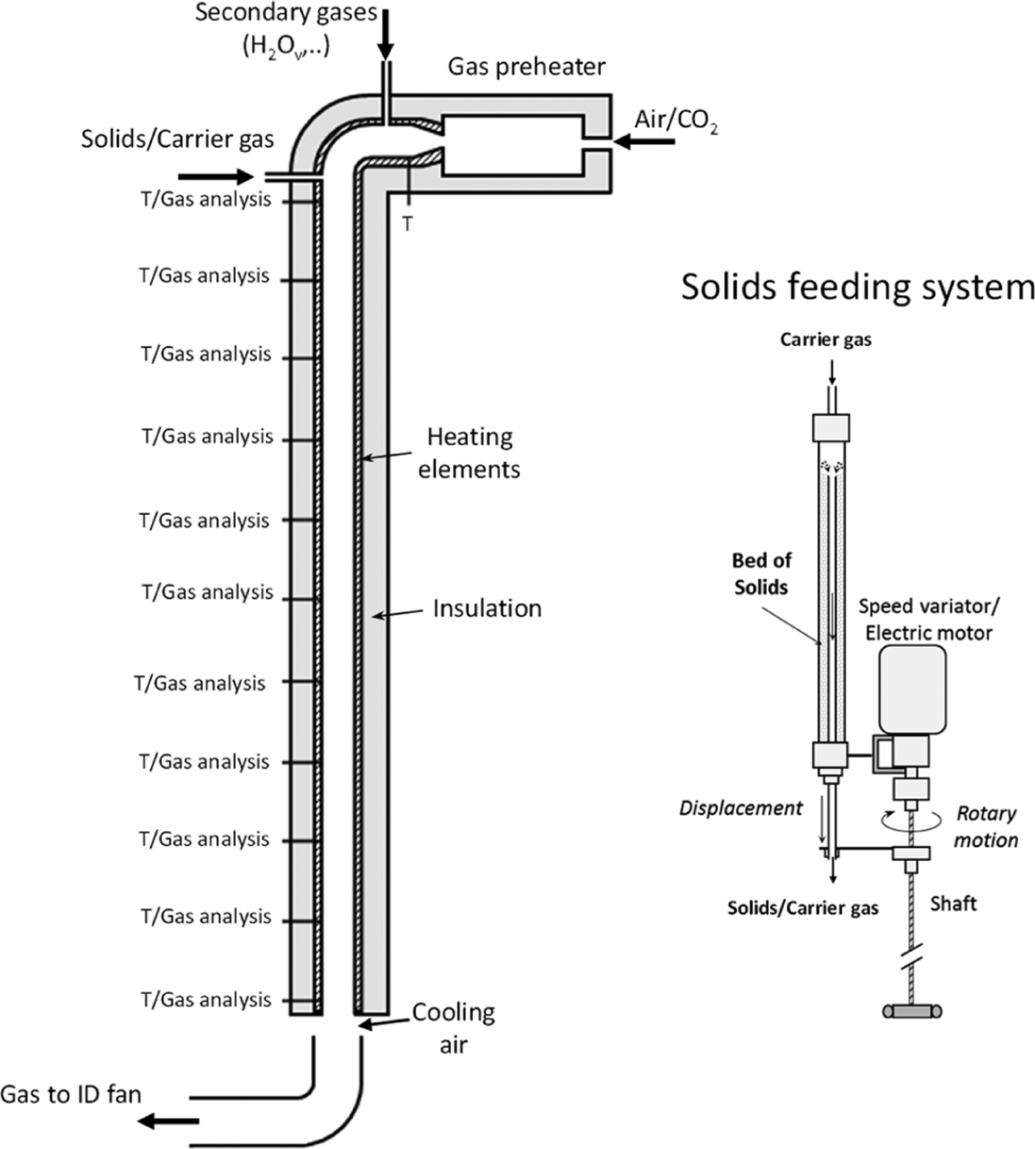
Scheme of the drop tube reactor (left) and solid feeding system (right) used during the Ca(OH)_2_ carbonation experiments.

The experiments were carried out in a drop tube reactor with a length of 5.2 m and an internal diameter of 0.08 m (see Figure 1). Main gas flows are heated up before being fed into the reactor using a 10 kW electrical preheater. In order to counteract heat losses and to maintain a uniform axial temperature profile, the reactor is equipped with three heating elements (of 3.5 kW each) connected to independent controllers to adjust the temperature in the different zones of the reactor. In addition, the reactor is isolated using glass wool, with a layer thickness of 0.2 m. There are 12 temperature measurement points at different heights that can also be used to measure the gas composition. Sorbent particles can be injected at the top of the carbonator (see Figure 1) to maximize the reactor length. The solid feeding system (schematically shown on the right-hand side of Figure 1) is composed by a cylindrical repository that contains the sorbent particles. Air is fed into the top of the repository which acts as a gas–solid carrier. Typically, an air flow of around 0.8 N m^3^/h is used during these experiments. A metallic drainage pipe (0.008 m i.d.) is located inside the bed of solids to drain them as the tip of the pipe moves downward. This pipe is fixed to a shaft that is connected to an electric motor equipped with a speed variator to control the motion. This system allows controlling the flow rate of solids as it is proportional to the vertical displacement velocity of the drainage pipe. In addition, a vibration device is attached to the cylinder to facilitate a uniform discharge of the sorbent. The pipe connecting the solid feeding system and the reactor is electrically heated and can be used to increase the temperature of the mixture of air and sorbent up to a maximum of 550 °C. Typically, a batch of around 150 g of Ca(OH)_2_ is loaded into the solid feeding system and flow rates of solids between 80 and 600 g/h were used during these experiments.

The synthetic flue gas used for the carbonation tests is composed of mixtures of air, CO_2_, and water vapor. Air is supplied from a blower and CO_2_ from compress gas cylinders. Flow rates of these gases are regulated using two mass flow controllers and mixed before being fed into the preheater. The water vapor is produced in a steam generator with a maximum capacity of 2.5 N m^3^/h. This is injected through an independent inlet into the reactor where it mixes with the preheated air and CO_2_. The experimental device is equipped with two gas analyzers (ABB EL3020 and ABB AO2000) to measure the gas composition at different heights during the experimental runs. In addition, a hygrometer (Dostmann P770) is used to measure the water concentration in the gas phase. All the measurements obtained from thermocouples, mass flow controllers, and gas analyzers are collected into a data logger for postprocessing. For these tests, commercial Ca(OH)_2_ was used as a sorbent whose main properties are reported in Table 1. As indicated in the Introduction section, this work focuses on the use of Ca(OH)_2_ as a sorbent in entrained carbonators; thus, the selected sorbent has a particle size of around 5 μm, typical of that used in in-duct sorbent applications.

**Table 1:** Main Properties of the Sorbent Used

	purity (% wt)	Dp_50_ (μm)	*S*_BET_ (m^2^/g)	density (kg/m^3^)	microporous volume (cm^3^/g)	average pore diameter (nm)
Ca(OH)_2_	93.3	5.2	14.5	2222	0.00043	223

In order to facilitate the interpretation of the results, these experiments have been carried out under differential conditions with respect to the gas phase by allowing only modest changes in the gas composition. Ideally, this ensures that all the sorbent particles react under very similar and controlled reaction conditions. A wide range of experimental conditions have been tested, as shown in Table 2. Regarding the particle residence time in the reactor, it has been assumed that the velocity of the solids along the reactor is given by the gas velocity considering the reduced gas/solid ratio and the low terminal velocity of the particles (<0.08 cm/s for the sorbent used in this work). Moreover, residence time distribution (RTD) experiments were carried out to characterize the gas flow patterns inside the reactor and to determine accurately the contact times between the gas and solids.

**Table 2:** Main Operation Conditions in the Drop Tube Reactor

	units	value
carbonation temperature	°C	300–650
inlet CO_2_ volume fraction	%_v_	0–25
inlet H_2_O volume fraction	%_v_	0–25
gas velocity	m/s	0.75–1.75
mass flow rate of solids	kg/h	80–600
particle residence time	s	<6

## Results and Discussion

**Figure 2:**
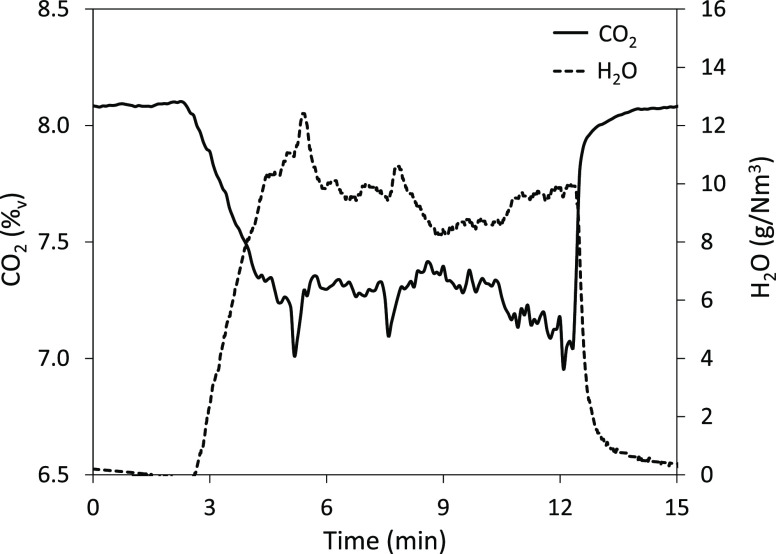
Evolution of the CO_2_ concentration and the increase in H_2_O in the gas phase during a typical Ca(OH)_2_ carbonation experiment (average carbonator temperature: 440 °C, gas velocity: 1.3 m/s, and mass flow rate of solids: 470 g/h).

An example of a typical carbonation experiment is shown in Figure 2. In this case, the CO_2_ (%_v_) and H_2_O (g/N m^3^) concentrations measured at the exit of the reactor are shown. Before starting the experimental run, the reactor is heated up and the different heating elements are adjusted to achieve a uniform temperature profile along the reactor. At the beginning of each test, there is no feeding of sorbent and the measurements of the gas analyzers are validated with the inlet mass flow rates of CO_2_ and air (including the air flow used to carry the solids into the reactor). In this particular experiment, the initial CO_2_ concentration is 8.1%_v_. Once the gas composition is stable, solids are injected into the reactor (at around minute 2.5 in Figure 2). This causes a reduction in the CO_2_ concentration and an increase in the H_2_O concentration in the gas phase. After an initial transition period, gas composition reaches a stable value of around 7.3%_v_ CO_2_ and 9.5 g H_2_O/N m^3^.

The small variations observed in gas composition are mainly due to fluctuations in the mass flow rate of solids. Then, after 10 min of operation under steady conditions, the feeding of solids is stopped and it is checked that the initial gas composition is reached to detect any air infiltration in the gas line of the analyzers or any malfunction with the gas supply system. For each experiment, the total amount of CO_2_ captured and H_2_O produced can be determined by the integration of these experimental curves in order to account for the small variations in the mass flow rate of solids. This allows us to determine the Ca conversion to CaCO_3_ (*X*_CaCO_3__) and Ca(OH)_2_ conversion to CaO (*X*_CaO_) as the amount of Ca(OH)_2_ fed during each test is known by weighting the solids at the beginning and at the end of each experiment.

**Figure 3:**
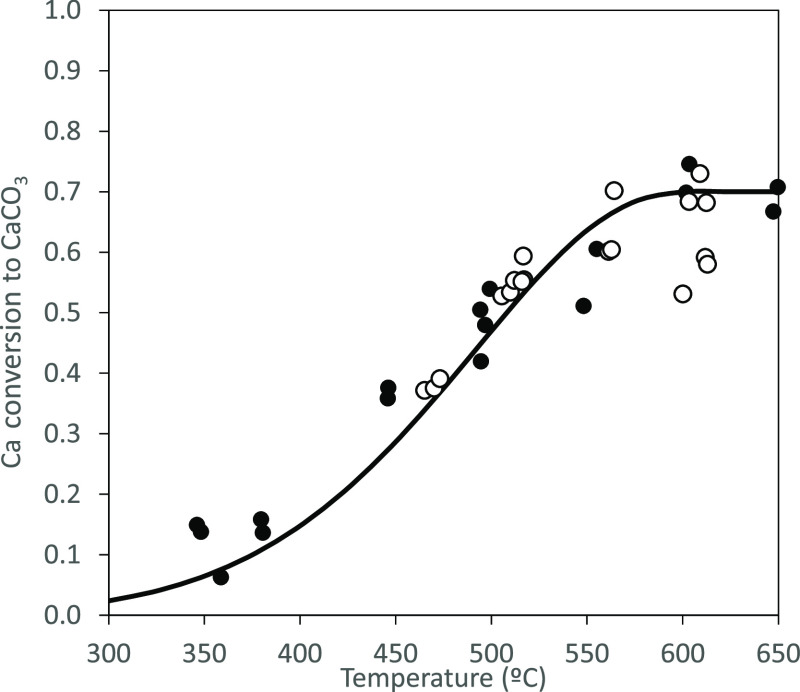
Effect of temperature on Ca conversion to CaCO_3_ (particle residence time 4 s, 7% CO_2v_). Solid symbols: no H_2_O in the reacting gases; empty symbols: 15%_v_ H_2_O in the reacting gases; and solid line: calculated values.

Several experiments were carried out to study the influence of the main variables affecting Ca(OH)_2_ carbonation (i.e., temperature, composition of the reacting atmosphere, and residence time). Figure 3 shows the effect of the carbonation temperature on the Ca conversion to CaCO_3_ (*X*_CaCO_3__). The results shown in this graph correspond to experiments carried out with a particle reaction time of 4 s and a CO_2_ concentration of 7%_v_. As can be seen, modest conversions of 0.15 are achieved at temperatures around 350 °C. However, it increases drastically with temperature, reaching an almost constant value of around 0.7 at temperatures above 600 °C. This conversion is typical of CaO derived from fresh calcined CaCO_3_. However, it is important to note the short reaction time used during this experiments that shows the fast carbonation kinetics of Ca(OH)_2_ compared to that corresponding to CaO which requires longer times (>30 s) to achieve a similar conversion under similar conditions. In this figure, there are also some experimental results marked as empty symbols that correspond to tests carried out with a 15%_v_ H_2_O in the reacting gas. As can be seen, similar conversions are achieved indicating that the presence of water has little influence on sorbent carbonation under the conditions tested in this work.

**Figure 4:**
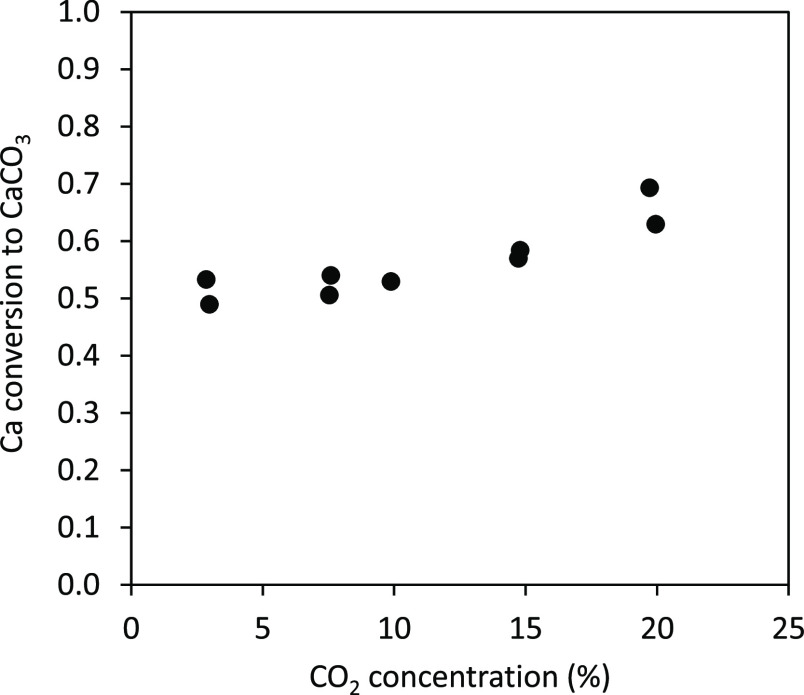
Effect of CO_2_ concentration on Ca conversion to CaCO_3_ (particle residence time 4 s and temperature 500 °C).

Following the composition of the reacting atmosphere, several experiments were carried out with different CO_2_ concentrations in order to evaluate its effect on Ca(OH)_2_ carbonation. As an example, Figure 4 shows the Ca conversion to CaCO_3_ under different CO_2_ concentrations for a reaction temperature of 500 °C and a reaction time of 4 s. As can be seen, this variable has limited impact on sorbent carbonation and only a moderate increase is observed for values higher than 15%_v_ CO_2_.

Plug flow of gases and solids was assumed in all tests reported above. Gas RTDs were carried out at 500 °C and 1.3 m/s gas velocity by means of step changes in the CO_2_ concentration (between 10 and 0%_v_ CO_2_) using a similar procedure as that followed in previous works (see refs (22) and (23) for more details). A dispersion number [*D*/(μL)] of 0.064 and a dispersion coefficient of 0.46 m^2^/s were determined for the reactor and conditions tested, which compares reasonably well with those obtained using the correlation proposed by Levenspiel (0.069 and 0.46 m^2^/s, respectively).^24^ This results in an actual residence time slightly lower than that estimated assuming an ideal plug flow reactor (PRF) pattern (*t*_r_/*t*_r,PRF_ = 0.96), which has been taken into account to slightly correct all experimental gas–solid contact times.

Based on the reaction mechanisms proposed in the literature,^25−28^ it has been assumed for the conditions tested in this work (i.e., short reaction times and small particle sizes) that the reaction proceeds through an initial decomposition of Ca(OH)_2_ (eq 1), followed by the carbonation of the formed nascent CaO (eq 2).
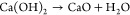
1
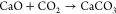
2
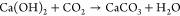
3

**Figure 5:**
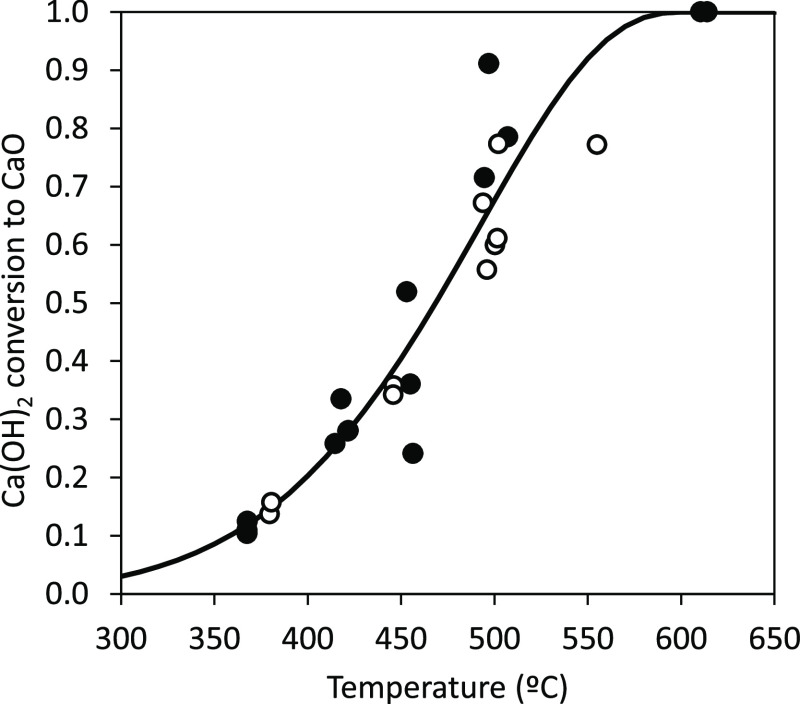
Effect of temperature on Ca(OH)_2_ conversion to CaO (particle residence time = 4 s). Solid symbols: dehydration experiments; empty symbols: carbonation experiments; and solid line: calculated values.

To elucidate which is the limiting step in the whole process (eq 3), several dehydration experiments were carried out to measure the Ca(OH)_2_ conversion to CaO (*X*_CaO_) and determine the reaction kinetics. Figure 5 shows the experimental conversion measured during dehydration experiments carried out in air for different reaction temperatures. As expected, temperature has an important effect on sorbent dehydration and full conversion can be achieved at temperatures above 600 °C after 4 s of reaction. This figure also includes the *X*_CaO_ measured during some carbonation experiments (empty symbols) thus in the presence of CO_2_. Similar values of Ca(OH)_2_ conversion to CaO have been measured during dehydration and carbonation experiments, indicating that CO_2_ has a negligible effect on sorbent dehydration.

**Figure 6:**
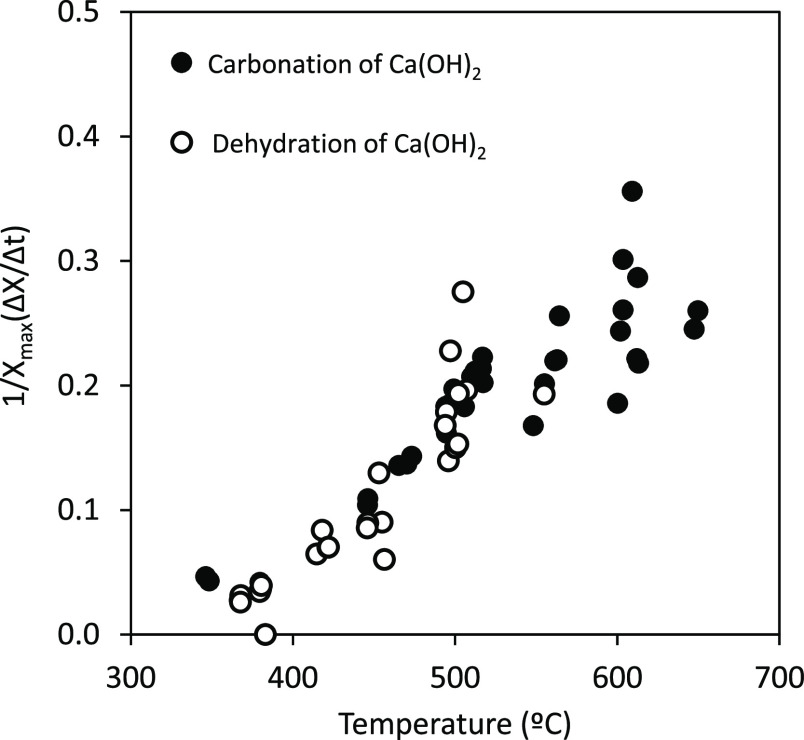
Comparison of the normalized reaction rates [1/*X*_max_(Δ*X*/Δ*t*)] for the Ca(OH)_2_ dehydration and carbonation. Empty symbols: dehydration step and solid symbols: whole carbonation process.

In order to compare the kinetics of the dehydration of Ca(OH)_2_ and the whole carbonation process, Figure 6 shows the normalized reaction rate with respect to the maximum conversion that can be achieved [1/*X*_max_(Δ*X*/Δ*t*)] (*X*_CaO,max_ = 1.0 for the dehydration reaction and *X*_CaCO_3_,max_ = 0.7 for the carbonation reaction). As can be seen, both reactions, Ca(OH)_2_ dehydration and the whole carbonation process, show similar normalized reaction rates indicating that the Ca(OH)_2_ dehydration is the rate-limiting reaction step. Moreover, these results also suggest that carbonation of the nascent CaO formed during dehydration can be considered an almost instant process with negligible effect on the kinetics of the whole Ca(OH)_2_ carbonation process.

According to this result, a simple approach has been followed to model the whole carbonation process which takes into account the kinetics of the initial Ca(OH)_2_ dehydration followed by the instantaneous conversion of the nascent CaO. A simplified shirking core model based on that proposed by Criado et al.^29^ assuming that the chemical reaction is the controlling step has been used to model the dehydration step.
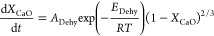
4

By integrating eq 4, the following expression can be obtained
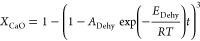
5

The pre-exponential factor (*A*_Dehy_) and the activation energy (*E*_Dehy_) have been calculated by fitting this equation to the experimental results. Values of 4359 s^–1^ and 63.2 kJ/mol have been obtained, respectively. This value of *E*_Dehy_ obtained agrees reasonably well with that reported by Criado et al.^29^ (60.8 kJ/mol) and the range of values reported in the literature (30–190 kJ/mol).^30^ As can be seen in Figure 5, the dehydration conversion can be predicted reasonably well with this model (solid line) and a good agreement with the experimental results can be observed.

Following the discussion mentioned above, once Ca(OH)_2_ conversion to CaO is calculated using eq 5, the carbonation conversion can be estimated assuming that the nascent CaO reacts with the CO_2_ present in the gas phase up to its maximum conversion
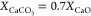
6

The solid line presented in Figure 3 corresponds to the carbonation conversion calculated using this methodology for different temperatures which present a reasonable agreement with the experimental results. Main differences are observed at temperatures below 400 °C, where the model tends to overpredict the carbonation conversion of the sorbent. This could be due to the negative effect the temperature has on the maximum carbonation conversion of CaO. Under these conditions, the assumption of eq 6 may overestimate the conversion of the nascent CaO. However, it is beyond the scope of this paper to study the Ca(OH)_2_ carbonation kinetics at low temperatures as these are not considered relevant for entrained reactors due to the maximum carbonation conversion achievable at *T* < 400 °C.^21,31,32^

**Figure 7:**
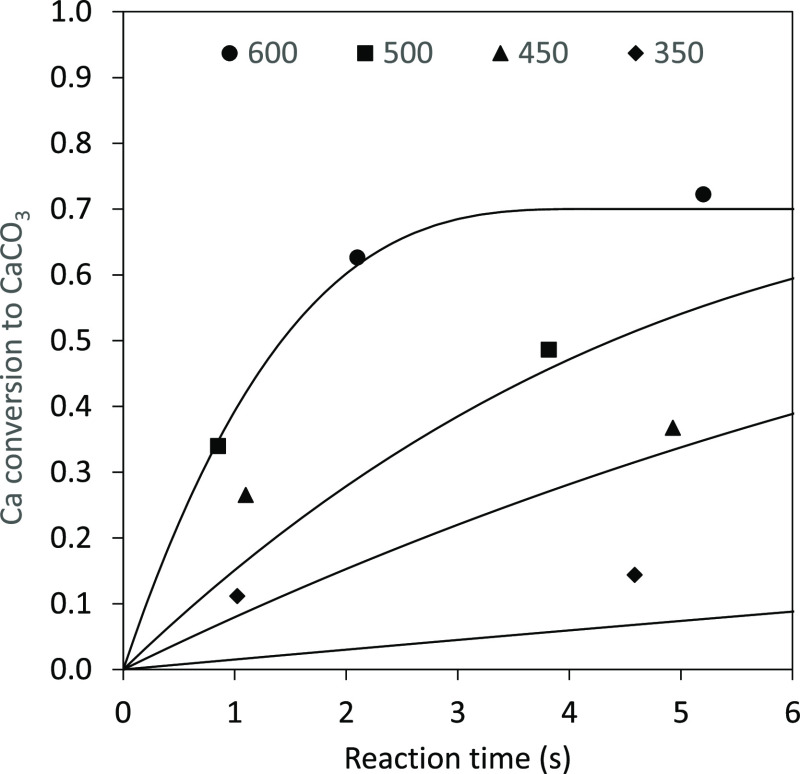
Evolution of the Ca conversion to CaCO_3_ with the reaction time for different carbonation temperatures (7%_v_ CO_2_). Symbols: experimental data and solid lines: calculated values.

Finally, Figure 7 shows the evolution of the Ca conversion to CaCO_3_ with the reaction time for different temperatures. As can be seen, *X*_CaCO_3__ values close to the maximum value can be achieved within 4 s at temperatures around 600 °C. The results presented in this figure confirm that high Ca(OH)_2_ carbonation conversions can be achieved under typical postcombustion conditions in reaction times of just a few seconds, thus supporting the viability of the use of entrained carbonator reactors for CO_2_ capture when Ca(OH)_2_ is used as a sorbent.

## Conclusions

The carbonation of Ca(OH)_2_ has been studied in a drop tube reactor under relevant conditions for postcombustion CO_2_ capture in entrained carbonator reactors. These experiments have been carried out using sorbent particles with an average particle size of about 5 μm. A wide range of experimental conditions with temperatures ranging between 350 and 650 °C and CO_2_ concentrations up to 25%_v_ have been tested. It has been demonstrated that carbonation conversions up to 0.7 can be achieved after 4 s of reaction time at temperatures above 600 °C. Results are consistent with a model of Ca(OH)_2_ carbonation proceeding through an initial dehydration of the sorbent followed by an almost instant carbonation of the nascent CaO formed. An activation energy for the dehydration step of 63.2 kJ/mol has been determined which is in agreement with those values reported in the literature. The results presented in this work should contribute to the design of entrained carbonator reactors and to the scaling up of CaL technology based on Ca(OH)_2_ as a sorbent.
